# MSC-Exos: Important active factor of bone regeneration

**DOI:** 10.3389/fbioe.2023.1136453

**Published:** 2023-02-06

**Authors:** Sihang Ren, Yuyang Lin, Wenyue Liu, Liqun Yang, Muxin Zhao

**Affiliations:** ^1^ Department of Plastic Surgery, The Second Hospital of Dalian Medical University, Dalian, China; ^2^ Department of Plastic Surgery, The First Hospital of China Medical University, Shenyang, China; ^3^ NHC Key Laboratory of Reproductive Health and Medical Genetics (China Medical University), Liaoning Research Institute of Family Planning (The Affiliated Reproductive Hospital of China Medical University), Shenyang, China; ^4^ Department of Biomaterials, Shengjing Hospital of China Medical University, Shenyang, China

**Keywords:** MSC-Exos, bone regenaration, mesenchyaml stem cells, osteogenic cells, biomaterials

## Abstract

Bone defect and repair is a common but difficult problem in restorative and reconstructive surgery. Bone tissue defects of different sizes caused by different reasons bring functional limitations and cosmetic deformities to patients. Mesenchymal stem cells (MSC), a major hotspot in the field of regeneration in recent years, have been widely used in various studies on bone tissue regeneration. Numerous studies have shown that the bone regenerative effects of MSC can be achieved through exosome-delivered messages. Although its osteogenic mechanism is still unclear, it is clear that MSC-Exos can directly or indirectly support the action of bone regeneration. It can act directly on various cells associated with osteogenesis, or by carrying substances that affect cellular activators or the local internal environment in target cells, or it can achieve activation of the osteogenic framework by binding to materials. Therefore, this review aims to summarize the types and content of effective contents of MSC-Exos in bone regeneration, as well as recent advances in the currently commonly used methods to enable the binding of MSC-Exos to the framework and to conclude that MSC-Exos is effective in promoting osteogenesis.

## 1 Introduction

Bone regeneration is an extremely complex repair process. The combination of cells, growth factors and structural framework is essential for bone regeneration (Battafarano et al., 2021). Bone repair is delayed or stopped if these three elements are affected or absent for various reasons within the individual with a bone defect. In adult individuals, a bone defect of 2 cm is the threshold value, and bone defects exceeding 2 cm are difficult to heal on their own without external forces. In order to break the limit, the study aims to fundamentally reconstruct the homeostasis of the three elements of bone regeneration and simulate the normal bone regeneration process *in vivo* (Sobacchi et al., 2013).

Mesenchymal stem cells are pluripotent non-hematopoietic stem cells with self-renewal capacity (Xia et al., 2019). MSCs are not only abundant, which can be found in bone marrow, adipose, muscle, peripheral blood, umbilical cord, placenta, fetal tissue, and amniotic fluid (Brown et al., 2019), but also involved in tissue repair, immunomodulation and anti-inflammation by controlling immune response, angiogenesis, cell proliferation, migration, invasion and survival (Heo et al., 2019). Previous studies suggested that MSC could be a source of cells in bone regeneration, but recent studies have shown that MSCs are not only the cellular basis for osteogenesis, but also parental cells for the release of various factors (Du et al., 2019). However, recent studies have shown that MSC is not only the cellular basis for osteogenesis, but also an activator and fusion agent for the release of various factors, or structural frameworks, making MSC an “all-rounder” in osteogenesis. This ability is fully reflected by the fact that exosomes concentrate the active components of MSC and stimulate the maximum osteogenic potential of MSC, while discarding the ethical issues, immune resistance (EL Andaloussi et al., 2013) and the inconvenience of excessive cell size (Verweij et al., 2019).

Exosomes are small vesicles secreted by cells that mediate intercellular communication through paracrine and other means (Schott et al., 2021). Exosomes are influenced by parental cells (Xia et al., 2019) and the carriage of these substances results in exosomes from different sources with specific and unchangeable parental characteristics (Wang et al., 2018a). Furthermore, spectrum-specific exosomes have a greater impact on the differentiation of MSCs than extracellular matrix, and osteoblast-derived exosomes containing osteogenic factors can reverse the lipogenic differentiation potential of MSCs cultured in lipogenic medium (Kargozar et al., 2019). These vesicles, which can be secreted by almost all living cells, are important carriers of information between osteoblasts and other cells, or between cells and the microenvironment (Peng et al., 2020). The secreted exosomes could be taken up by recipient cells *via* endocytosis, ligand-receptor interaction (where both the ligand and the receptor are not clearly uncovered) or fusion then the contained bioactive cargos are transferred to modify gene expression, signaling, and overall functions and behaviors of recipient cells. Artificially interfered with exosomes can contain RNA, enzymes, therapeutic genes or drugs and other contents (Basso and Bonetto, 2016). Therefore, the effective application and potential development of MSC-Exos is an important topic and a necessary tool in the field of bone regeneration. By reviewing the various contributions of MSC-Exos in osteogenesis in recent years, we acknowledge the significant contribution of MSC-Exos in osteogenesis and also find the limitations and defects due to its low yield, difficulties in purification or difficulties in targetin.

## 2 Effect of MSC-Exos on osteogenesis-related cells and active factors

**FIGURE 1:**
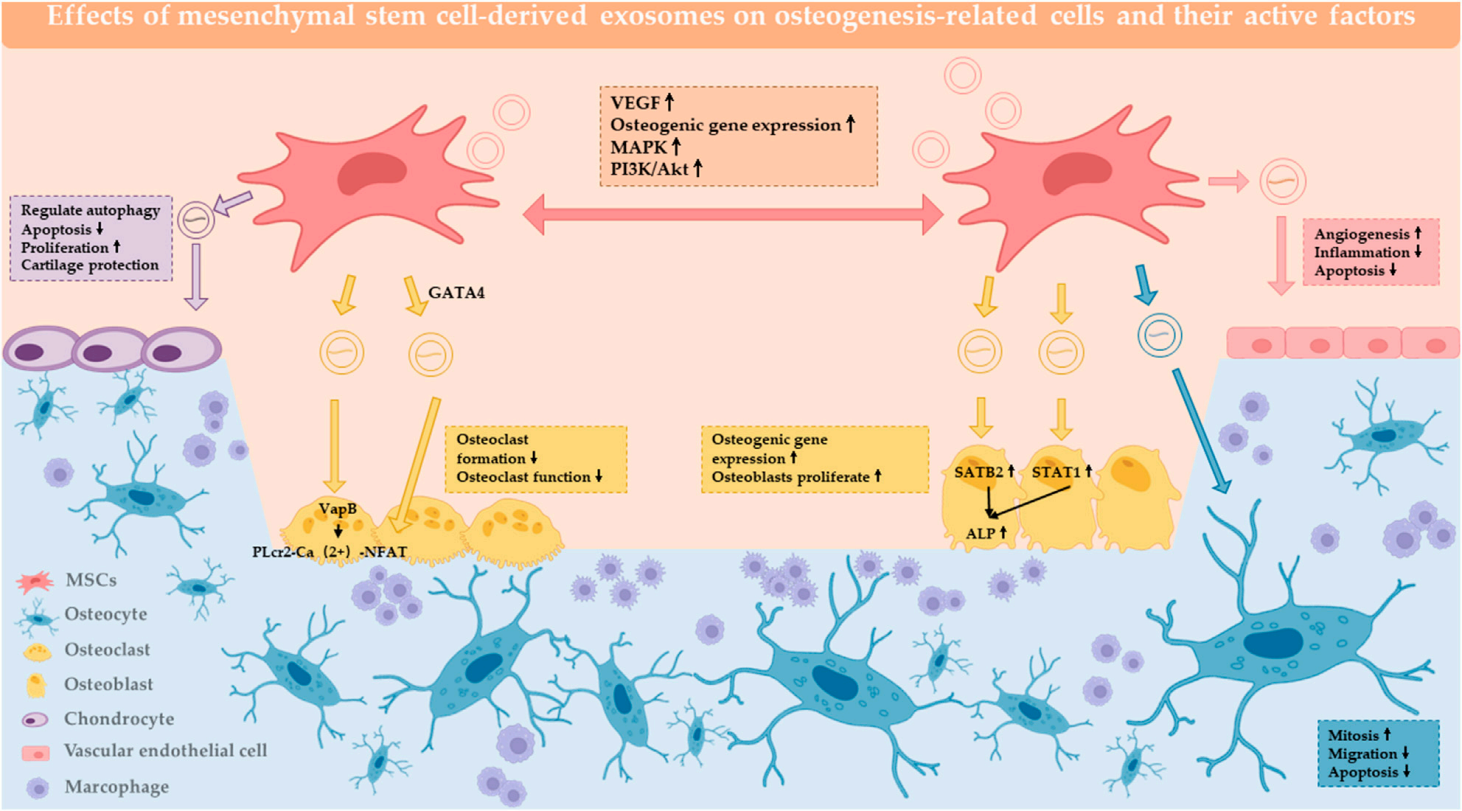
Effect of MSC-Exos on osteogenesis-related cells and active factors. MSC-EXOs act on MSCs, osteoblasts, osteoclasts, chondrocytes, osteocytes, endothelial cells, and the local immune microenvironment to have a positive impact on bone regeneration.

In the process of osteogenesis, cellular components are the basis of the whole process, including osteoblasts, osteoclasts, osteocytes, and chondrocytes (Kim and Mikos, 2021). The positive effect of MSC exosomes on the proliferation and/or migration of multiple cell types *in vitro*, including osteoblasts, osteocytes, MSCs and endothelial cells (Jia et al., 2020). The Effects of mesenchymal stem cell-derived exosomes on osteogenesis-related cells and their active factors are summarized in Table 1; Figure 1. Activation of the core cells can effectively promote the initiation and rapid progression of the osteogenic process (Li et al., 2018). In turn, the effect on macrophages provides beneficial regulation of the inflammatory environment of bone regeneration (Schlundt et al., 2021). Therefore, it can be assumed that the primary effect of MSC-Exos on cells lies in the direct action on osteogenic cells (Liu et al., 2021a). The active factors of the osteogenic elements are considered to be more important components than cells. In conjunction with the trend of being “cell-free”, the effective use of osteogenic factors and tissue frameworks plays a key role.

**TABLE 1:** Effect of exosomes derived from mesenchymal stem cells on osteogenic associated cells.

Source	Target cells	Effect	Reference
jaw-BMSC	MSC	upregulate maxillary BMSC-ilium osteogenic ability	Li et al. (2019)
hPDSCs		promote hADSC osteogenic differentiation	Jin et al. (2020)
hGMSC		bringing miR-296 and miR-210 to promote bone regeneration of calvaria defects, associated with vascularization increasing	Pizzicannella et al. (2019a), Pizzicannella et al. (2019b)
hPDLSC			Zhai et al. (2020)
hMSCs		bringing osteogenic miRNAs to induce MSC differentiation towards osteoblasts	Yang et al. (2019)
BMSC	Osteoblasts	bringing LncRNA MALAT1 to promote SATB2 expression (silencing of SATB2 is able to reduce ALP activity in osteoblasts)	Zhang et al. (2021a)
BMSC		bringing miRNA-935 to promote osteoblast proliferation and differentiation	Wang et al. (2019), Wang et al. (2021a)
BMSC-Exo with KCNQ1OT1 knockdown		inhibit the block of primary osteoblast proliferation	Wang et al. (2021a)
lnc-KCNQ1OT1-modified ADSCs-Exos		attenuate cytotoxicity and apoptosis of TNF-α-induced primary osteoblasts	Hu et al. (2021)
BMSC-Exo	Osteoclasts	bringing miRNA-335 to inhibit osteoclast differentiation and stabilizes fracture	Yang et al. (2020a)
OMSC-Exo		bringing miR-206-3p to inhibit osteoclastogenesis and promote osteogenic differentiation	Guo et al. (2021a)
MSC	Chondrocytes	enhance chondrocytes migration, proliferation, chondrogenic differentiation and matrix synthesis ability	Otsuru et al. (2018), Zhang et al. (2018)
infrapatellar fat pad (IPFP) MSCs-derived exosomes		bringing miR-100-5p to regulatedchondrocyte autophagy, thereby inhibiting apoptosis	Wu et al. (2019)
hiPS-MSC		bringing miRNA-135b to romote cell proliferation and inhibit apoptosis	Zhang et al. (2020)
MSC		attenuate inflammation and restoring matrix homeostasis in alleviating TMJ osteoarthritis	Zhang et al. (2019)
MSC	Osteocytes	anti-apoptotic effects onosteocytes and bone marrow MSCs	Yin et al. (2017)
LLLI-ADSC-exo		inhibits hypoxia-induced apoptosis and promotes bone defect repair	Zhu et al. (2017)
PSC-derived EVs		suppress osteocytes shRNA expression	Xu et al. (2019a)
ADSC	endothelial cells	bringing miRNA125a to promote the formation of endothelial tip cells and angiogenesis	Liang et al. (2016)
ADSC		bringing miRNA-21 to promote endothelial cell vascularization	An et al. (2019)
ADSC		bringing miRNA-21 and miRNA322 to promote angiogenesis	Kato et al. (2021)
HUVEC-Exo		bringing miRNA-423-5p to promote proliferation, migration and tube formation	Xu et al. (2019b)
ADSC		bringing LncRNA SNHG9 to inhibiting the inflammatory response and apoptosis, upregulate endothelial cell function	Song et al. (2020)
MSC	Macrophages	reduce the pro-inflammatory marker expression and ROS production	Zhang et al. (2022)
MSCs-Exo derived form orofacial bone		bringing miR-223 and miR-let-7c to promote polarization to M2 macrophages	Yuan et al. (2017)
ADSC	T cells	bringing miR-20a to promote the differentiation of Th17 and Treg from naive CD4^+^ T cell	Bolandi et al. (2020)

### 2.1 MSC-Exos act on MSC

Information transfer between MSCs through MSC-Exos is the initial direction in MSC-Exos research (Zhu et al., 2019). When jaw BMSC-Exos was co-cultured with iliac BMSC, it was found that ALP and osteogenic gene expression were upregulated, while siRNA against Rab27a blocked this osteogenic effect, and it was concluded that Exo interacted between BMSC-J as well as BMSC-I to upregulate maxillary BMSC osteogenic ability (Li et al., 2019). When human dental pulp stem cells (hPDSCs) were co-cultured with human stem cells (hADSC), the phenotype of osteoblast was upregulated and was able to upregulate the MAPK pathway, leading to phosphorylation of ERK1/2 as well as JNK, thus promoting hADSC osteogenic differentiation (Jin et al., 2020). MSC-derived sEVs increase the expression of VEGFA and VEGFR2 in MSCs and thus may facilitate osteogenesis and angiogenesis of bone regeneration (Pizzicannella et al., 2019a; Pizzicannella et al., 2019b). MSC-Exos induces osteogenic differentiation by using its cargo. hADSC-Exo, which is predifferentiated in osteogenic medium, may act on different targets of osteogenesis by altering its miRNA expression profile at different stages of culture and induce MSC differentiation towards osteoblasts by activating different pathways including PI3K/Akt as well as MAPK and thus initiate osteogenesis and promote bone regeneration (Zhai et al., 2020).

### 2.2 MSC-Exos act on osteoblasts

BMSC-Exo is able to carry the LncRNA MALAT1 and act as a sponge for miR-34c to promote SATB2 expression, while silencing of SATB2 is able to reduce ALP activity in osteoblasts and mineralized nodules (Yang et al., 2019). In osteoporotic rats, BMSC-Exos-miRNA-935 was able to act on osteoblasts to regulate STAT1 pathway, promoting ALP synthesis and osteoblast proliferation and differentiation, thereby inhibiting osteoporosis progression in OVX rats (Zhang et al., 2021a). After TNF-α acts on primary osteoblasts, it can upregulate the expression of miRNA-141-5p, thereby inhibiting osteogenesis. As a sponge, lncRNA-KCNQ1OT1 inhibits the block of primary osteoblast proliferation by miRNA-141-5p. ADSC-lncRNA-KCNQ1OT1 acts as the sponge of miRNA-214, and inhibits its expression, thereby upregulating BMP2 expression and promoting osteogenic differentiation of BMSC (Wang et al., 2019). In addition, lncRNA-HOTAIR targeting miRNA-138 and lncRNA-LOXLT-AS1 targeting miRNA-196a-5p have been found to play a regulatory role in osteogenesis (Wang et al., 2021a).

### 2.3 MSC-Exos act on osteoclasts

BMSC-Exos-miRNA-335 acts on VapB (vesicle associated membrane protein-associated protein B), and inhibition of its expression can target activation of the Wnt/β-catenin pathway and promote fracture recovery. VapB is a key target for regulating PLcr2-Ca ^2+^-NFAT signaling, and inhibition of this signaling inhibits osteoclast differentiation and stabilizes fracture ends (Hu et al., 2021). The Wnt pathway is a classical pathway of osteogenesis. ADSC-Exo-miRNA-130a-3p mediates the SIRT7/Wnt/β-catenin axis, targets silencing of SIRT mRNA, and activates the Wnt pathway, thereby preventing β-catenin from being degraded and entering the nucleus completes its process of promoting osteogenic gene expression (Yang et al., 2020a). The orofacial MSC appears to be unique in orofacial skeletal development. OMSCs are derived from neural crest cells (NCCs). The results confirmed that miRNA-206-3p is an important downstream factor of GATA4 (GATA-binding protein 4: transcription factor that regulates the expression of RUNX2 and TGF-β). Under the action of GATA4, upregulates miRNA-206-3p, promotes NFATc1 (nuclear factor of activated T cell cytoplasm 1) expression and suppresses BMP3 expression, thereby inhibiting osteoclastogenesis and promoting osteogenic differentiation (Guo et al., 2021a).

### 2.4 MSC-Exos act on chondrocytes

Chondrocytes treated with MSC-derived small extracellular vesicles (sEVs) enhanced migration, proliferation, chondrogenic differentiation and matrix synthesis ability (Otsuru et al., 2018; Zhang et al., 2018). This cascade of responses induced by MSC-derived sEVs leads to the stimulation of multiple cellular responses, such as cell survival, proliferation, differentiation and migration, which facilitated tissue repair (Chew et al., 2019). Recently, it was reported that targeting mTOR signaling in chondrocytes mediated by MSC-derived sEVs as an alternative mechanism for chondroprotection. The results showed that MSC-derived sEVs downregulated mTOR signaling, and regulated IL-1-mediated chondrocyte autophagy, thereby inhibiting apoptosis and regulating cellular metabolism for matrix production (Wu et al., 2019). In a mouse OA model induced by IL-1β and collagenase, MSC-Exos was able to promote COL2A1 and proteoglycan expression and resist IL-1β-induced chondrocyte proliferation inhibition and apoptosis. Further study revealed that lncRNA-KLF3-AS1 and miRNA-206 are competitive endogenous RNAs (ceRNAs), and miRNA-206 can promote the expression of GIT1 (G-protein coupled receptor kinase interacting protein 1) lncRNA-KLF3-AS1 acts as a sponge for miRNA-206 and inhibits the expression of miRNA-206, thus achieving a protective effect against cartilage damage. In addition, hiPS-MSC-Exo acts on the PDCD4 (programmed cell death protein 4) gene *via* miRNA-135b, activating the downstream caspase-3 pathway, promoting OCN expression, promoting cell proliferation and inhibiting apoptosis (Zhang et al., 2020). MSC-Exos alleviate TMJ osteoarthritis by attenuating inflammation and restoring matrix homeostasis through the mechanism of Adenosine, a receptor for receptor cells, binds to the CD73 ligand on the surface of the MSC-Exos membrane, causing the release of Exo contents into chondrocytes and activating the Akt/ERK/AMPK pathway, thereby regulating matrix homeostasis (Zhang et al., 2019).

### 2.5 MSC-Exos act on osteocytes

MSC exosomes have anti-apoptotic effects on osteocytes and bone marrow MSCs, producing proliferation-promoting, migration-promoting, anti-apoptotic effects on MSCs after low-intensity laser irradiation (Yin et al., 2017). ADSC-secreted Exo (LLLI-ADSC-Exo) inhibits hypoxia-induced apoptosis and promotes bone defect repair (Zhu et al., 2017). Human perivascular stem cell (PSC)-derived EVs promote mitosis, cell migration, and suppress osteoblast shRNA expression through tetra-transmembrane proteins to achieve pro-osteogenic effects (Xu et al., 2019a).

### 2.6 MSC-Exos act on endothelial cells

ADSC-Exo-miRNA125a is able to bind to the 3′UTR target of delta-like4 (DDL4), thereby promoting the formation of endothelial tip cells and angiogenesis (Liang et al., 2016). Also, ADSC-miRNA-21 acts on the PTEN pathway to promote endothelial cell vascularization, while exerting a similar effect (An et al., 2019).

Meanwhile, bone regeneration repair in a rat cranial defect model was achieved by PLGA/PDA scaffold mounted with Exo in the experiment *in vivo*. Under the action of GW4869, ADSC-Exo promoted the entry of ADSC-exo-miRNA-21, miRNA-27b, miRNA322, and let-7i into endothelial cells through the ESCRT-independent pathway, where miRNA-21 inhibited PTEN as well as Smad7 pathway, and miRNA-322 inhibited Cul-2 pathway, thus promoting angiogenesis (Kato et al., 2021). Therapeutic effect of human umbilical cord MSC exosomes in osteonecrosis, attributing the anti-apoptotic and pro-survival effects of MSC exosomes to miR-21-mediated downregulation of PTEN (phosphatase and tensin homolog) and AKT (protein kinase B) phosphorylation (Kuang et al., 2019). In a model of hindlimb ischemia, ADSC-Exo promotes M2 polarisation, inhibits iNOS expression and time-as well as dose-dependently upregulates arginase 1 (Arg-1) levels, in which miRNA-21 plays a major role and inhibits Akt phosphorylation as well as CSF-1 secretion, and through this mode of action, promotes vascular regeneration in the ischaemic limb (Zhu et al., 2020). MiRNA-423-5p acts on the sufu of HUVECs, promoting their proliferation, migration and tube formation (Xu et al., 2019b). BMSC-Exos promotes HUVEC proliferation, migration and regulates the Hippo pathway through the YAP target, promoting angiogenesis, and by transporting lysophosphatidic acid (LPA) and autocrine motility factors (ATX) also act on the Hippo pathway, prompting YAP/TAZ in complex with TEAD to direct nuclear gene expression and induce cartilage reconstruction in temporomandibular joint osteoarthritis (Wang et al., 2021b). LncRNA SNHG9 enters endothelial cells *via* ADSC-Exo and then targets and inhibits TRADD mRNA (TNF-R1-associated death domain), causing TRADD protein degradation, inhibiting the inflammatory response and apoptosis, thereby upregulating endothelial cell function (Song et al., 2020). Angiogenesis is a critical step in osteogenesis and a key target for the treatment of ischemic bone diseases.

### 2.7 MSC-Exos act in the osteoimmune microenvironment

Osteoimmunology reveals that the immune system and the skeletal system are closely linked and share many commonalities in terms of cytokines, receptors and signaling. Immune cells often play the role of regulators in the formation of the local bone microenvironment: by regulating the expression of various factors, they regulate osteogenic differentiation, osteolytic differentiation, fibrosis, vascularisation and other processes closely related to bone regeneration (Schmidt-Bleek et al., 2014; Chen et al., 2016). The release of regulatory factors by immune cells can influence the osteogenic and osteolytic processes in bone tissue. Macrophages play an important role in the regulation of the bone immune microenvironment. The different subtypes of macrophages correspond to the stages of fracture healing. In the inflammatory phase, activated M1 macrophages perform phagocytosis and produce pro-inflammatory cytokines such as TNF, IL-1β, IL-6 and IL-12 to promote early and mid-stage osteogenesis. In the late phase, alternative activated M2 macrophages release pro-regenerative cytokines such as IL-10, TGF-β, BMP2, and VEGF to establish an anti-inflammatory environment and promote osteochondral differentiation and angiogenesis (Shin et al., 2021). Zhang et al. (2022) developed bioactive 3D PLA-Exo scaffolds and found significantly lower expression of pro-inflammatory markers and reactive oxygen species (ROS) compared to the PLA scaffold group. After co-culture of exosomes from jawbone-derived OMMSCs with macrophages, increased miR-223 in macrophages could be achieved by inhibiting Pknox1 expression, polarizing macrophages toward the M2 type (Yuan et al., 2017). It has also been shown that ADSC-Exos containing miR-10a promotes the differentiation of naive CD4^+^ T cells towards Th17 and Treg (Bolandi et al., 2020).

## 3 MSC-Exos in combination with biomaterials for bone defect repair

**FIGURE 2:**
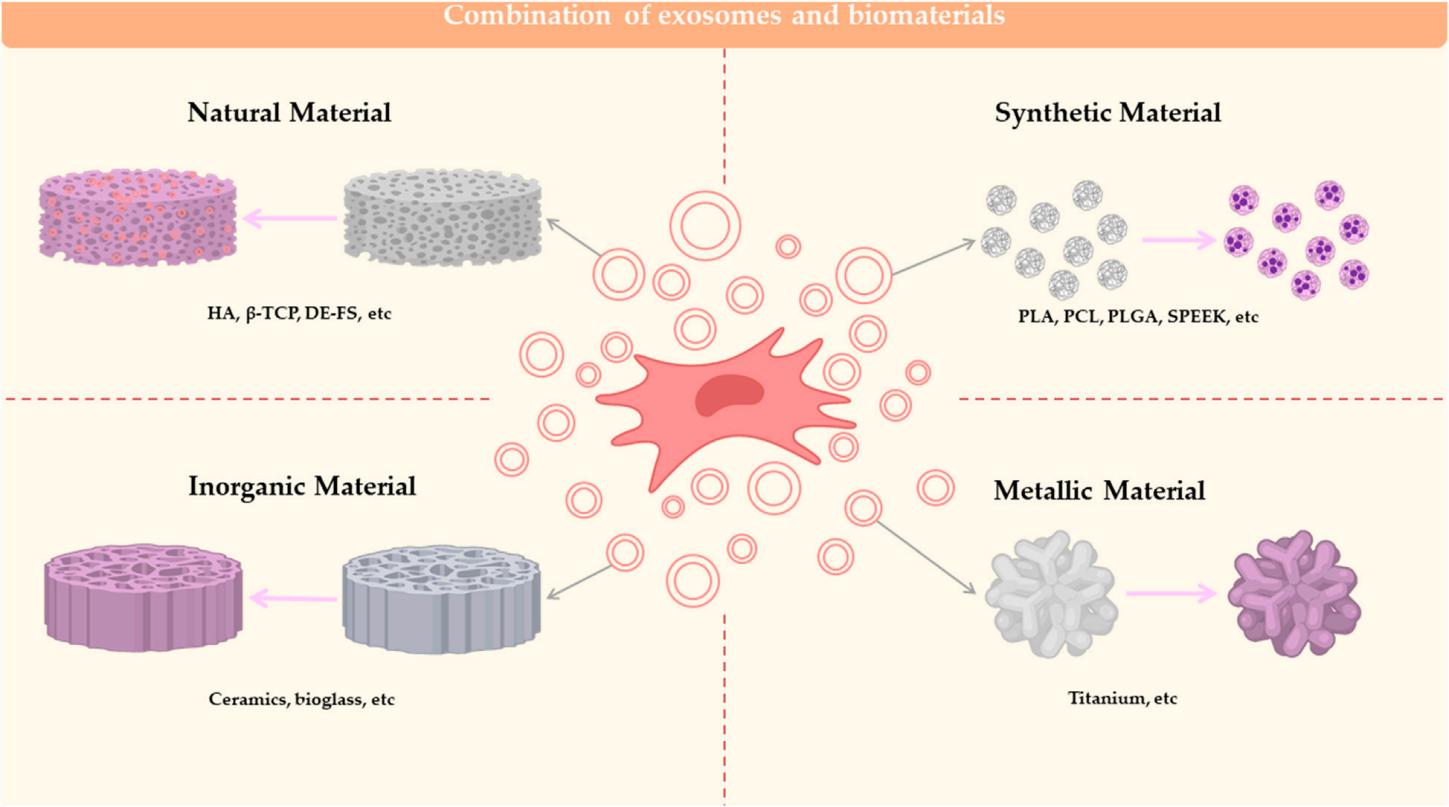
Combination of exosomes and biomaterials. Biomaterials combined with MSC-EXO for bone defect repair can be classified into 4 categories: natural material, synthetic material, inorganic material and metallic material.

Currently, most bone defects are repaired with bone replacement materials (Carriel et al., 2018), which are not optimally suited to replacement due to their biological inertness, foreign body reactions and the additional damage caused after placement. The combination of biomaterials with MSC-Exos enables adhesion between cells and implants and is now used as a method for *in vivo* experiments in scientific research in the direction of osteogenesis, not only for testing the osteogenic effect of MSC-Exos, but also for clinical transformation. MSC-Exo is mostly combined with biological material by co-incubation. Among the biomaterials, the more mature materials can be divided into natural polymers, synthetic polymers, metal and inorganic non-metal materials (Ren et al., 2022a). The Classification of biomaterials combined with MSC-EXOs are summarized in Figure 2.

### 3.1 Natural material

#### 3.1.1 Hydroxyapatite scaffold

Pouya et al. synthesized and characterized hydroxyapatite (HA) scaffold, following the osteogenesis and angiogenesis of HA scaffold with or without endometrial mesenchymal stem cells (hEnSCs) derived exosomes were investigated in rat with calvaria defect (Youseflee et al., 2022). Studies have shown that Alg (alginate)/HA is inferior to Alg/hydroxyapatite (Hap) in terms of mechanical properties, cytocompatibility and induction of cell differentiation in MSC (Yu et al., 2017). Therefore, some studies have chosen to use periumbilical stem cell-Exo co-embedded with hydroxyapatite (HAP) in HA-Alg for bone regeneration (Yang et al., 2020b). Some studies have also used this Exo combined with novel coral hydroxyapatite (CHA), filamentous protein (SF), glycolic chitosan (GCS) and bifunctional polyethylene glycol (DF-PEG) to form a novel self-healing hydrogel, which was successfully used for the repair of bone defects in mice (Wang et al., 2020a). MSC-Exos wrapped hydrogels form MSC-Exos-HAGel + nanohydroxy apatite-poly (hHP) to form uMSCEXO/Gel/nHP, which promotes angiogenesis by promoting endothelial cell proliferation and migration. In-depth studies have found that miRNA-21 is the target for intercellular communication and promotes the notch/DLL4 pathway to achieve pro-angiogenic purposes (Bohner et al., 2020).

#### 3.1.2 β-tricalcium phosphate

β-tricalcium phosphate (β-TCP) is resorbable, osteoconductive and osteoinductive, making it one of the most potent bone graft substitutes (Zhang et al., 2016). Exosomes can enhance the osteoinductivity of β-TCP by activating the PI3K/Akt signaling pathway in hBMSCs, which implies that the exosome/β-TCP scaffold has better osteogenic activity than the β-TCP scaffold (Ying et al., 2020). And BMSC-Exoss-HIF1α combined with β-TCP scaffolds could repair critical-sized bone defects by promoting new bone regeneration and neovascularization (Nikhil and Kumar, 2022).

#### 3.1.3 Composite natural scaffold

Aman et al. prepared two types of cryogels, namely chitosan-gelatin-chondroitin sulfate (CGC) for articular cartilage and nanohydroxyapatite-gelatin (HG) for subchondral bone (Bahar et al., 2022).A novel bilayer cryogel manufactured using a single process of two layers (CGC as top layer and HG as the bottom layer) to mimic osteochondral units. The CS/HA/Exo combination is a new therapy for bone defect repair to induce bone formation. The CS scaffold significantly promoted bone regeneration compared to the control (Wang et al., 2022).

#### 3.1.4 Others

Exosomes derived from osteogenic differentiated BMSC (OBMSC) are osteogenic, leading to the proposal of a novel exosomal decellularized fish scale (DE-FS) scaffold for promoting bone regeneration *in vivo*. The DE-FS scaffold is obtained through a process of decellularization and decalcification and has high biocompatibility and low immune rejection. The intrinsically anisotropic structure of DE-FS enhances the adhesion and proliferation of BMSCs *in vitro* (Zhang et al., 2022).

### 3.2 Synthetic material

#### 3.2.1 PLA scaffolds

Zhang et al. developed bioactive 3D PLA scaffolds based on exosomes to enhance their osteogenic and immunomodulatory potential (Zhang et al., 2022). PLA (polylactic acid)-10CaSi (calcium silicate) was produced by the thermal phase separation technique Porous scaffolds were placed in HBSS and immersed for 28 days. The surface micromorphology of the scaffolds was studied by ESEM-EDX and the exo-rich complex was used for bone regeneration with good results (Gandolfi et al., 2020). Zha et al. (2021) used ATDC5-derived exosomes to encapsulate the VEGF gene and construct gene-activated engineered exosomes. The specific exosome anchor peptide CP05 was used as a flexible joint to effectively combine engineered exosome nanoparticles with 3D-printed porous bone scaffolds. It was also verified that the CP05 anchor peptide (PCL-CP05)-modified 3D printed PCL scaffold could effectively induce massive vascularized bone regeneration.

#### 3.2.2 PCL scaffolds

Chondroprogenitor cell-derived (ATDC5) exosome plays a key role in osteogenic induction and vascular remodeling of large segmental bone defects by transfecting VEGF plasmid and binding to GPI, CP05 and CD63 to form a composite 3D scaffold material of ATDC5-Exo-VEGF-GPI CP05 using PCL as a scaffold (Zha et al., 2021). In addition, S-GSNO and MSC-Exos-modified PCL, GSNO modulates fibrin structure, limits Plt activity, inhibits coagulation and thrombosis, and GSNO alters blood coagulation structure, thus providing a critical contribution to early blood scab formation and early bone regeneration (Wang et al., 2020b).

#### 3.2.3 PLGA scaffolds

Li et al. (2018) demonstrated the development of a polydopamine-encapsulated PLGA scaffold to achieve controlled release of hASC-Exos and repair of cranial defects in mice. Gao et al. adsorbed hypoxic Exosomes onto the surface of injectable porous poly (lactide-co-glycolide) (PLGA) microspheres with bioinspired polydopamine (PDA) coating (PMS-PDA microspheres) to induce vascularized bone regeneration in 5-mm rat calvarial defect (Gao et al., 2022). Swanson et al. (2020) improved encapsulation and controlled release of poly (lactic acid-glycolic acid) (PLGA) and poly (ethylene glycol) (PEG) triblock copolymer microsphere exosomes on a tunable time scale.

#### 3.2.4 Sulfonated polyetheretherketone scaffolds

Bone marrow stem cell (BMSC)-derived exosomes contain a variety of signaling molecules and have been proven to have immunomodulatory functions. Fan et al. (2021) developed a BMSC-derived Exos functionalized implant that accelerates osseointegration through immunomodulation. BMSC-Exoss is reversibly incorporated onto tannic acid (TA)-modified sulfonated polyetheretherketone (SPEEK) *via* strong interactions of TA with biomolecules. The slow release of exosomes from SPEEK can be phagocytosed by co-cultured cells, which can effectively improve the biocompatibility of SPEEK.

### 3.3 Inorganic non-metallic materials

Sr replaces Casi ceramics into BMSC to promote endocytosis of Exo-carrying miRNA-146a by HUVECs, allowing miRNA-146a to promote angiogenesis by inhibiting Smad4 and Nf2 pathways in target cells. This alternative approach greatly reduces rejection through the release of anti-inflammatory factors, and is of great clinical application (Liu et al., 2021b). The use of lithium-containing glass ceramics co-cultured with BMSC produced Li-BGC-BMSC-Exos, which highly expresses miRNA130a, promotes endothelial cell proliferation, migration and angiogenesis by inhibiting PTEN protein expression, as well as Akt activation (Liu et al., 2019). In addition, ionic products from bioglass acting on MSC were found to promote the expression of nSMase2 as well as Rab7a, thereby promoting endothelial cell proliferation, migration and angiogenesis by promotion of exosomes genesis and the ability to increase the content of miRNA-1290 and decrease the content of miRNA-342-5p in the output exosomes and promote the expression of vasoactive factors such as VEGF, thus promoting angiogenesis. This study, although focusing on novel biomaterials, provides constructive insights into the mechanisms of Exo product action in promoting Exo genesis (Zhang et al., 2016). BMSC and ADSC from different sources were screened for BMSC-Oi-Exo in osteoinduction medium or normal medium with increased levels of miRNA-328a, miRNA31a-5p, let-7c-5p, and let-7c-5p, and activation of BMP through miRNA regulation of BMP induction by BMPR2/Acvr 2b Smad1/5/9 phosphorylation. The Exo lyophilized formulation is also bound to the material by binding to a hierarchical porous bioactive glass scaffold to achieve maintenance of bioactivity and slow release (Liu et al., 2021c).

### 3.4 Metal scaffolds

Due to the limited duration of Exo activity *in vivo*, some studies have shifted the focus to maintaining the long-term stability and osteogenic ability of Exo *in vivo*. It was found that the combination of Ti-pyrrole -biotin forms a Bio-ppy-Ti complex, followed by the addition of streptavidin (SA), with ADSC-evs. The combination forms a novel biomaterial that exerts an efficient and stable bone-enabling effect (Chen et al., 2019). Alternatively, lyophilization techniques were used to address the problems of low cell implantation rate and short cell life span during Ti cage osteoinduction (Bari et al., 2021). A complex Gr-Ti scaffold was prepared using ADSC-derived Exos. The results showed that the Gr-Ti scaffold had low toxicity and good biocompatibility and promoted the adhesion and osteogenic differentiation of ADSCs. Exosomes play a role in promoting osteogenic differentiation of ADSCs: mRNA levels of RUNX2, ALP, and Osterix were significantly higher in the Gr-Ti/Exos group than in the Gr-Ti group, and this process was associated with the Wnt signaling pathway. Gr-Ti scaffolds with ADSC and ADSC-derived Exos successfully repaired mandibular defects in rabbits. The bone density and flexural strength were significantly higher in the Gr-Ti/Exos group than in the Gr-Ti group (Sun et al., 2022).

## 4 Regulators of MSC-Exos production

Exosomes are tiny EVs as their small yields hinder the expansion of basic research in exogenous analysis. Intracellular calcium levels, external stress, cytoskeletal blockade, drug effects, gene expression factors and other factors all affect EXOs production (Liu and Su, 2019). Therefore, it is of great significance for its practical application in the future to increase its output through the engineering production of exosomes.

### 4.1 MSC pretreatment

**FIGURE 3:**
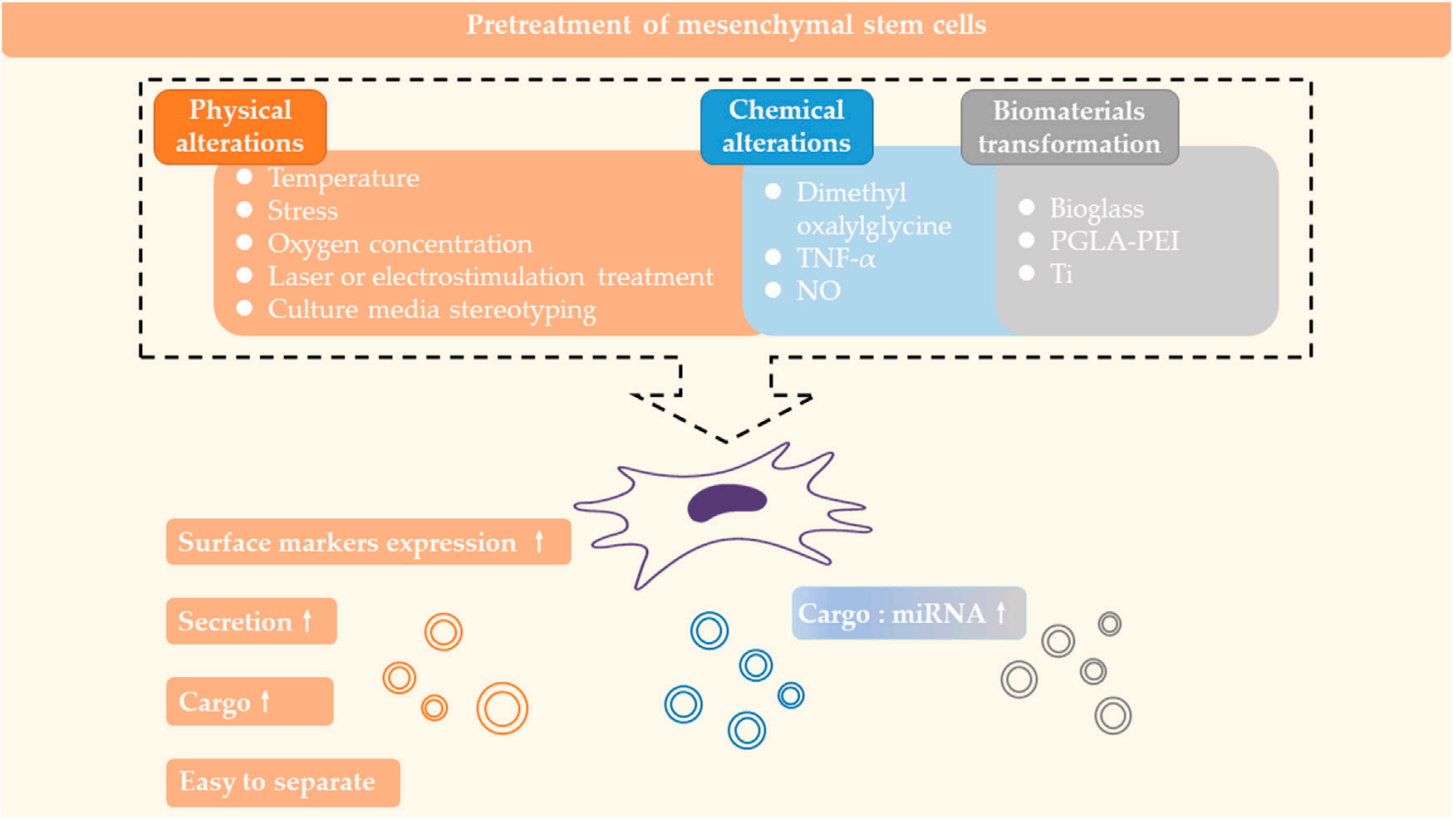
Pretreatment of mesenchymal stem cells. Physical and chemical alterations of MSCs or cultivation of MSCs with kinds of biomaterials can affect the formation, secretion and carrying of exosomes.

The characteristics of exosomes vary with the source of MSCs. When the MSC is subjected to different external effects, it transmits different signaling factors to exosomes to guide exosomes to work. Thus, treatment of the MSC can influence the formation, secretion and cargo of the exosome from the initiation. The Pretreatment of mesenchymal stem cells are summarized in Figure 3.

#### 4.1.1 Physical alterations

##### 4.1.1.1 Temperature

By changing the ambient temperature of cultured cells (42°C and 37°C), it was found that the surface markers CD9 and CD63 were more significantly expressed in the 42°C group. And that bone regeneration experiments using heat stress-induced ADSC-Exo with β-TCP (tricalcium phosphate) as a carrier were more effective than using β-TCP alone or using the ADSC with β-TCP (Akdeniz-Dogan et al., 2021).

##### 4.1.1.2 Stress

BMSC under cyclic mechanical stress (CMS) regulates osteogenic differentiation *via* HDAC1 and Dnmt 3b epigenetically, while RUNX2-sensitive miRNA-103a is upregulated by stress in BMSC, and CMS-BMSC-Exo inhibits osteoclast formation and function by suppressing the NF-kB pathway, in the same way that acting on periodontal membrane exosome inhibits IL-1β production in M, thereby suppressing apoptosis and inhibiting bone formation (Xiao et al., 2021). The rotary cell culture system (RCCS) provides a novel mechanical environment for HUMSCs, and the different speeds of RCCS can increase exosome production within 196 h at the beginning of culture, and RCCS acts on HUMSCs through the microgravity and fluid shear of the RCCS, resulting in high expression of lncRNA-H19. lncRNA-H19 is a highly conserved sequence involved in stem cell formation and function. is a highly conserved sequence involved in stem cell differentiation, embryonic growth and tumorigenesis. This highly expressed lncRNA H19 is released *via* exosomes and acts on chondrocytes to promote chondrocyte proliferation, anti-apoptosis, ECM formation and function (Yan et al., 2021).

##### 4.1.1.3 Culture media stereotyping

Bioreactors act on PDSCs, ADSCs, and SMSCs within 2D or 3D culture media through fluidic and tensile forces, and act on Yes-associated-protein (YAP) through mechanical stimulation, thereby activating the Wnt pathway and promoting the secretion and production of osteogenic exo (Guo et al., 2021b). Dental pulp pluripotent stem cells (DPPSC) are considered to be an important source of stem cells in the field of bone regeneration, and 3D culture promotes Nanog expression and osteogenesis, as well as the isolation of exosomes, providing a serum-free environment that excludes the influence of serum-derived exosomes on experimental result (Faruqu et al., 2020).

##### 4.1.1.4 Oxygen concentration

Hypoxia-pretreated ADSC-Exo was able to promote angiogenesis in adipose grafts thereby promoting ADSC as well as adipose graft survival (Han et al., 2018). Using MLO-Y4 as a model, the environment of hypoxia and serum deprivation triggered increased apoptosis by ROS. hADSC-Exo was able to inhibit RANKL expression, thereby suppressing osteoclast-mediated osteoclastogenesis, and by upregulating Bcl-2/Bax, inhibiting ROS and reducing cytochrome enzyme production, thereby resisting apoptosis in the hypoxic environment (Ren et al., 2019). It has even been suggested that Evs in hypoxic environments are important factors in regenerative medicine. This effect is induced by HIF-1α, which hydroxylates and cleaves HIF-1α in a normoxic environment *via* prolyl hydroxylases (PHDs) on proline residues. In contrast, under hypoxia, PHDs require oxygen in conjunction with α-ketoglutarate and Fe, so HIF-1α hydroxylation is reduced and stabilized and translocated in the nucleus, activating hypoxia-sensitive genes that regulate cell cycle, apoptosis and cell differentiation (Imanirad et al., 2014). In contrast, MSC under hypoxic conditions exhibit reduced Mit activity, increased glucose depletion, reduced Mit autophagy, reduced ROS, lower telomeric shortening rates, and decreased cellular senescence overall (Noronha et al., 2019). Therefore, the influence of hypoxia has been incorporated in various studies on MSC-Exos and the hypoxic state of animal models or cultured cells has been achieved by atmospheric hypoxia, chemical hypoxia or other methods to interfere with the mechanism of action of MSC-Exos.

##### 4.1.1.5 Laser or electrostimulation treatment

After low-intensity laser irradiation, it produced proliferation-promoting, migration-promoting and anti-apoptotic effects on MSC (Yin et al., 2017). Exosomes secreted by ADSC (LLLI-ADSC-Exo) was able to inhibit hypoxia-induced apoptosis in osteoblasts 40 and promote bone defect repair (Wang et al., 2018b). In contrast, after radiation treatment of hPMSC, miRNA-23a levels in extracted Exo decreased, thereby inhibiting the CXCL12 (chemokine 12) pathway in BMSC and thus inhibiting BMSC osteogenesis (Zhuang and Zhou, 2020). ESCRT-III-related protein Alix stimulated ADSC in the microgrooved matrix, promoting secretion of EVs, upregulation of miRNA pro-angiogenesis, and increased secretion of growth factors (Ji et al., 2021).

#### 4.1.2 Chemical alterations

Dimethyl oxalylglycine-stimulated human bone marrow mesenchymal stem cell-derived exosomes enhance bone regeneration through angiogenesis by targeting the AKT/mTOR pathway (Liang et al., 2019). Inflammatory preconditioning of ADSC-Exo has immunosuppressive properties (Domenis et al., 2018). Pretreatment of ADSC with TNF-α, which mimics the acute inflammatory microenvironment, promotes Wnt3a expression and enhances osteogenic gene expression in ADSC-CM by pretreating parental cells with Exo (Lu et al., 2017). Alternatively, Exo production was promoted by TNF-α acting on human dental pulp MSCs carrying miRNA-1260b, which inhibited osteoclastogenesis due to Wnt5a and promoted M2 polarization, thereby inhibiting periodontal inflammatory bone loss (Nakao et al., 2021). In addition, it has been shown that controlled Nitric Oxide releasing acts on PMSC to promote exo-miRNA-126 release as well as VEGF expression and acts on EC to promote angiogenesis and indirectly promote osteogenesis (Du et al., 2017).

#### 4.1.3 Biomaterials transformation

The use of lithium-containing glass ceramics co-cultured with BMSC produced Li-BGC-BMSC-Exo with high expression of miRNA130a, which promoted endothelial cell proliferation, migration and angiogenesis by inhibiting PTEN protein expression, as well as Akt activation (Liu et al., 2019). In addition, ionic products in bioglass acting on MSC were found to promote the expression of nSMase2 as well as Rab7a, thus promoting Exo output by promoting Exo genesis, and were able to increase the content of miRNA-1290 in the output Exo, decreasing the content of miRNA-342-5p, and promote the expression of vasoactive factors such as VEGF, thus promoting angiogenesis, the study, while focusing on novel biomaterials, also provided constructive insights into the mechanisms of Exo promotion by the action of ionic products (Bohner et al., 2020). The composite particles made by using PLGA and PEI as coating materials and cyanine 5.5 as an indicator, in which SPIO magnetic nanoparticles were encapsulated, promoted the secretion of Exo under dynamic-induced endocytosis and were able to carry a variety of miRNAs including miRNA-2127, achieving antioxidant, regeneration, proliferation and immune (Park et al., 2020). M-Exo isolated under BMP2 stimulation was co-cultured with nanotube implants and was able to promote osteogenesis (Wei et al., 2019). It was shown that there are two structures of micro/nanostructured hierarchical titanium topographies, reticular and tubular, both of which can extend the diffusion area of BMSC on the Ti surface and promote BMSC osteogenesis *in vitro*, with the key targets being SMPD3 (sphingomyelin phosphodiesterase 3) and Rab27b (the small GTPase Rab27), which promotes Exo biogenesis and secretion to improve osseointegration (Zhang et al., 2021b).

### 4.2 MSC-Exos pretreatment

Exosome engineering summarises the common methods used regarding Exo treatment, such as freeze-thaw cycles, sonication, electroporation, extrusion, click chemistry, antibody, etc (Choi et al., 2021; Khayambashi et al., 2021). In contrast to the osteogenic modification of MSC-Exo, current research is more inclined to develop new ideas and methods while verifying the effectiveness and safety of the original methods.

It has been shown that ADSC-Exo proteins obtained by different isolation methods are characterized differently. Studies comparing ultracentrifugation (UC), size exclusion chromatography (SEC), Exo Quick-TC precipitation and Exo Quick-TC ULTRA isolation methods found that all proteins were involved in integrin pathways and inflammatory pathways, but the UC method also involved in cholecystokinin-R (CCKR) signaling pathway, ROS, and angiogenesis; qEV favored CCKR, EGF-R, and cytoskeletal regulation; TC favored cytoskeleton, angiogenesis, EGF-R, and PDGF-R; and Tcu appeared to be similar to TC but with a slightly lesser role for PDGF-R and concluded that different isolation methods of ADSC-Exo had different effects on LPS-stimulated cells differently, and if studies in related fields are conducted, the TCU method is recommended to promote greater tube formation function (Huang et al., 2021).

The modification of the exosome can be divided into two aspects: cargo filling and exosome membrane modification, but of course, not being bound to natural sources of exosomes, one also sets out to reassemble the components of the exosome thus engineering the production of exosomes.

#### 4.2.1 Cargo filling

The role of MSC-Exo is largely dependent on the modulation of target cells and environments by the active factors carried by its contents, therefore enrichment of the contents in the exosome will be central to enhancing the osteogenic role of the Exo.

##### 4.2.1.1 Electroporation

With regard to Exo modification, electroporation is the more common practice, using a PBS solution to dilute the exosome extracted from MSC and then mixing it with the target contents in an electroporation buffer. After electroporation, the electroporation solution is aspirated and placed in a new RNase-free Eppendorf tube and kept at 37°C for 30 min to restore the integrity of the exosome membrane. Subsequently, the solution was centrifuged at 10,000 × g for 2 h to remove excess target contents (Duan et al., 2021). However, it was found that membrane defects in the Exo triggered by electroporation were still difficult to repair in a short period of time, promptly by resting, thus triggering adverse effects on the structural integrity and function of the Exo (Khayambashi et al., 2021). However, it has been shown that electroporation can inactivate exo-let-7 in preosteoblasts, thereby inhibiting osteogenesis (Liu and Su, 2019).

##### 4.2.1.2 Freeze-dried

The use of freeze-dried ADSC-Exos and GMP-compliant pharmaceuticals can be used to modulate immunity (Choi et al., 2019). And the freeze-dried method is generally not used alone, but mostly in combination with biological materials. For example, in experiments with GMSC-Exos to promote wound healing, chitosan solution was mixed with silk fibroin solution and then the chitosan-silk fibroin emulsion was incubated at −20°C for 12 h, then at −70°C for 6 h and then lyophilized in a vacuum freeze dryer for 48 h to obtain a chitosan/silk fibroin hydrogel sponge that was effective for wound healing (Bari et al., 2019).

##### 4.2.1.3 Freeze-thaw cycles

A mixture of exosome and liposome is designed using membrane fusion of the exosome and liposome. Exosomes and liposomes are prepared separately and then mixed in equal volume ratios. the mixture is frozen in liquid nitrogen and thawed at room temperature for 15 min. The freeze-thaw cycle is repeated several times and the resulting mixture is a mixture of exosomes and liposomes. This method allows for the carriage of membrane surface proteins and markers and membrane fusion (Shi et al., 2017).

#### 4.2.2 Membranes modification

##### 4.2.2.1 Click chemistry

Atomic transfer radical polymerization for the packaging of Exo has also been successful (Sato et al., 2016), with PLGA (polylactic acid-ethanolic acid copolymer) + PEG (polyethylene glycol) forming triblock copolymer microspheres and assembling Exo by droplet microfluidics to form a controlled mineralized Exo, which is combined with the aqueous and organic phases to make an Exo scaffold, thus making a cell-promoting craniofacial bone regeneration free material 75. A study combined HUVEC-Exo with new coral hydroxyapatite (CHA), filamentous protein (SF), glycolic chitosan (GCS) and bifunctional polyethylene glycol (DF-PEG) to make a composite hydrogel to promote bone regeneration. It was found that the new composite had more new bone tissue and morphogenetic protein 2 (BMP-2) and the highest microvascular density 61. Another study used exosome-encapsulated dexamethasone sodium phosphate (Dex) nanoparticles (Exo/Dex) with a folic acid (FA)-polyethylene glycol (PEG)-cholesterol modified (Chol) compound on its surface to achieve an active targeted drug delivery system (Lathwal et al., 2021).

##### 4.2.2.2 Receptor-ligand

After the study selected bone marrow stromal cell target bone (ST)-derived Exo (Ste-Exos) and clarified its characteristic action of promoting BMSC osteogenesis, ste-exos was modified to improve its difficulty in improving postmenopausal osteoporosis in OVX mice after intravenous administration *in vivo*, and the targeted delivery of Exo was achieved through the assembly of Ste-Exos and aptamer complexes to form a complex targeting BMSC (Yan et al., 2020).

##### 4.2.2.3 Electrostatic interaction

The anionic environment in the joint cavity results in minimal utilization of MSC-Evs in intra-articular injectables. A study used a new cationic amphiphilic macromolecule: e-polylysine-polyethylene distearyl phosphatidylethanolamine to modify MSC-Evs, reversing their surface charge, and the method maintains the integrity of the Evs with no interference with the cargo, resulting in good stability in the presence of anionic macromolecule interference (Luo et al., 2019).

##### 4.2.2.4 Hydrophobic insertion

IL-10 pretreatment of immature DC (imDC) induces tolDex, the Dex that inhibits inflammation in osteoarthritis. The study used tolerogenic DC (tolDC)-derived exosome to immobilise ROS-sensitive thioredoxin (TK)-embedded polyethylene glycol (PEG) joints on the exosome surface using a hydrophobic insertion method. This processing method was used to achieve targeted delivery of the exosome to mature DCs, reduce CD40 expression levels and counteract the inflammatory response of osteoarthritis. In addition, the presence of PEG was found to prolong the duration of action of the exosome in the circulation as well as in inflamed joints (Feng et al., 2021a).

#### 4.2.3 New attempts

Some studies have even artificialized Exo, using nanoporous membranes, microfluidics and other techniques to extract cell fragmentation, adding specific exosome cargo and designing a mimetic of Exo for replacing biological sources of Exo that are less abundant and have more uncontrollable factors in the organism (Lee et al., 2021).

Combined with the current research progress, based on the improvement of each key step of MSC-Exo, and the trend of mainstream research ideas, we can roughly deduce the scheme of exo optimization: firstly, to provide a 3D culture environment for MSC and to encapsulate the active ingredients of MSC-Exo by modifying the contributing bone factors, to select a suitable vector for the specificity of different sites of bone defects The vector is selected for the specificity of the bone defect at different sites, and the target binding of Exo is enhanced by means of receptors and ligands to promote the maximization of Exo function. In the future, researchers may need to integrate multiple approaches to develop standardized and consistent quality procedures and propose methods for large-scale production of MSC-Exo.

## 5 Future perspectives

When we look for clinical applications of bone regeneration biomaterials, we are used to evaluating them in terms of safety, biocompatibility, the difficulty of preparation, price and convenience of use, so it is particularly important to build an evaluation system for biomaterials for bone defect repair.

The combination of exosomes and biomaterial scaffolds while maintaining the activity of the exosomes is the challenge of this technology, which has been solved by hydrogels, lyophilization and surface modification of the scaffold material. Recent literature has focused on keywords such as 3D bioprinting, composite scaffolds and multi-porous scaffolds, which may reveal future research hotspots in this field. However, it is important to notice that we should not stiffly composite exosomes and scaffolds at the expense of the properties of both themselves.

The advantages of combining exosomes with biological scaffolds are: 1) the enrichment of exosomes; 2) the storage of exosomes; 3) the avoidance of ethics due to “cell-free”, which is the current research trend and facilitates clinical translation. Although exosomes have a promising application in bone tissue repair, there are also many constraints that hinder their development, as they are difficult to extract, with low yields, low target utilization, and often difficult to act accurately in complex *in vivo* environments. The existing research focuses on 1) the mechanism of MSC-Exos osteogenesis in different sources and environments, and 2) the modification and functional enhancement of MSC-Exos.

As for materials, they should not only act as a carrier, but should intervene at all stages of exosome production. For example, combining different properties of materials to promote the secretion of exosomes containing active ingredients from parental cells, or the isolation or accumulation of exosomes through the adsorption of materials, and also researchers have focused on how to achieve functional substitution of exosomes through the exploration of lipophilic materials, thus enabling the mass production of exosomes.

Although MSC-Exos were promising therapeutic agents, the research on the efficacy of MSC-Exos on bone regeneration is still in its infancy. Numerous *in vitro* trials have demonstrated the effectiveness of MSC-Exo in bone disease, (Feng et al., 2021b), while most experiments *in vivo* have been conducted on small animals (including rats, mice and rabbits), validating the effectiveness of MSC-Exo on bone regeneration in animal models of bone defects and diseases such as osteonecrosis and osteoporosis (Tan et al., 2020), supporting the basis for clinical translation of MSC therapeutic agents (Ren et al., 2022b). However, the clinical trials of MSC-Exo is still limited. www.clinicaltrials.gov lists 7 clinical trials of exosomes in relation to bone, 3 of which describe the use of exosomes as an early diagnostic tool and 2 for clinical treatment. One is attempting to verify whether transplantation of hASCs-CM and a synthetic bone substitute can facilitate maxillary bone repair, and another is attempting to verify the feasibility of intradiscal injection of PRP with exosomes derived from blood for the treatment of chronic low back pain but has not yet published results. As the translation of MSC-Exo-based therapies from preclinical studies to the clinic requires a number of key parameters, including the establishment of optimal MSC culture conditions and exosome production, isolation and storage protocols to provide batch-to-batch consistency, optimal dosing and exosome dosing schedules, and the development of potency assays to allow efficacy evaluation (Mendt et al., 2019). More clinical trials remain to be conducted.

In addition, we should also focus on enhancing the compliance of MSC-Exosomes and the immunomodulatory effects of MSC-Exosomes on bone regeneration. Bone regeneration is not only about bone defects caused by trauma and fractures, but also about bone loss caused by systemic diseases such as osteoporosis, femoral necrosis or drugs. Therefore, it is not enough to combine exosomes with biomaterials, but also to focus on the concentration enhancement and targeted presentation of exosomes in circulation, and thus to maximize the contribution of exosomes to the three basic elements of bone regeneration.

## 6 Conclusion

The production of MSC-Exos can be achieved by direct action on various osteogenic cells or by carrying substances that affect cellular activity factors or the local internal environment in target cells, or by binding to biological materials to activate the osteogenic framework. The generation of MSC-Exos can be achieved by pretreatment of its parental cells and exosome contents and membranes with a view to obtaining the possibility of mass production of MSC-Exos.
